# Socio-Economic Predictors and Distribution of Tuberculosis Incidence in Beijing, China: A Study Using a Combination of Spatial Statistics and GIS Technology

**DOI:** 10.3390/medsci6020026

**Published:** 2018-03-21

**Authors:** Gehendra Mahara, Kun Yang, Sipeng Chen, Wei Wang, Xiuhua Guo

**Affiliations:** 1 Department of Epidemiology and Health Statistics, School of Public Health, Capital Medical University, No.10 Xitoutiao, Youanmen Wai Street, Fengtai District, Beijing 100069, China; gbmahara@gmail.com (G.M.); yangkun1123@163.com (K.Y.); ivanchen2010sp@hotmail.com (S.C.); 2 NAMS, Bir Hospital, Kathmandu 44600, Nepal; 3 Beijing Municipal Key Laboratory of Clinical Epidemiology, Beijing 100069, China; 4 Department of Evidence-Based Medicine, Xuanwu Hospital, Xicheng District, Beijing 100053, China; 5 Fuwai Hospital, Chinese Academy of Medical Science, Xicheng District, Beijing 100037, China; 6 School of Medical Sciences, Edith Cowan University, Perth, Joondalup WA6027, Australia

**Keywords:** tuberculosis, socio-economic factors, spatial statistics, Beijing, China

## Abstract

Evidence shows that multiple factors, such as socio-economic status and access to health care facilities, affect tuberculosis (TB) incidence. However, there is limited literature available with respect to the correlation between socio-economic/health facility factors and tuberculosis incidence. This study aimed to explore the relationship between TB incidence and socio-economic/health service predictors in the study settings. A retrospective spatial regression analysis was carried out based on new sputum smear-positive pulmonary TB cases in Beijing districts. Global Moran’s I analysis was adopted to detect the spatial dependency followed by spatial regression models (spatial lag model, and spatial error model) along with the ordinary least square model were applied to examine the correlation between TB incidence and predictors. A high incidence of TB was seen in densely populated districts in Beijing, e.g., Haidian, Mentougou, and Xicheng. After comparing the R^2^, log-likelihood, and Akaike information criterion (AIC) values among three models, the spatial error model (R^2^ = 0.413; Log Likelihood = −591; AIC = 1199.76) identified the best model fit for the spatial regression model. The study showed that the number of beds in health institutes (*p* < 0.001) and per capita gross domestic product (GDP) (*p* = 0.025) had a positive effect on TB incidence, whereas population density (*p* < 0.001) and migrated population (*p* < 0.001) had an adverse impact on TB incidence in the study settings. High TB incidence districts were detected in urban and densely populated districts in Beijing. Our findings suggested that socio-economic predictors influence TB incidence. These findings may help to guide TB control programs and promote targeted intervention.

## 1. Introduction

Tuberculosis (TB) is a chronic infectious bacterial disease caused by *Mycobacterium tuberculosis*, which mostly affects the lungs, although it can affect other organs and parts of the body. It is a curable and preventable disease, which is transferred via air (through droplets of a cough or saliva of actively infected patients) [1,2]. Furthermore, there are several risk factors for TB disease for example; low immune status, poor living conditions, unhealthy and unbalanced diet, presence of disease (diabetes and HIV/AIDS), and behavior factors; tobacco smoking [3]; alcohol consumption [4]; demographic factors (population density, migration, and social instability); and socio-economic status (employment, poverty, per capita income gross domestic product (GDP), and GDP at a district level) [5,6,7,8,9,10,11] as reported in earlier studies [1,12,13,14,15]. Beijing, one of the most populated cities in the world, has more than 21 million permanent residents and over 7 million migrants residing there [16]. In recent years, China has experienced rapid economic development and urbanization, due to the movement of the migrant population into urban areas like Beijing from rural parts of country, which is contributing to increase prevalence and incidence of TB infection [16,17]. It has already been established that frequent travelers from rural areas to urban cities such as Beijing (floating population) are more likely to transmit the disease along the way [18]. 

Several studies have been conducted earlier regarding influencing factors contributing to TB incidence and prevalence in China; however, such studies in the Beijing region are lacking. In addition, multivariate regression models including linear regression [19], logistic regression [20], negative binomial regression [9] and partial least square path modeling [13] were used in those studies [7,21]. It is difficult to incorporate with the spatial heterogeneity of the effects of ecological, geographical and socio-economic factors from those traditional models [13,22]. Very few studies have applied the GIS technique for TB characteristics investigation so far [7,21]. The recently developed GIS technique is the preferred method and technique for studying epidemic features of an infectious diseases [21], as it allows easier and more efficient acquisition, data visualization, manipulate, analyze and display of the geographical data from other traditional methods for disease clusters [23]. This GIS application technology is rapidly being applied to investigate the disease prediction and distribution when baseline data are unavailable or difficult to access [24].

Accordingly, investigating the disease clusters and the role of spatial heterogeneity in the relationship between affecting factors and TB incidence is essential for TB control and prevention strategy development. Thus, this study aimed to investigate the socio-economic predictors and distribution of tuberculosis based on TB notification data in Beijing using spatial regression analysis. 

## 2. Materials and Methods

### 2.1. Study Area and Design

A retrospective ecological study was conducted in 16 districts in the Beijing region based on the reported new (sputum smear-positive) pulmonary tuberculosis cases from 2005 to 2014. Beijing lies on the northern tip of the North China plain at 39°56′ N and 116°20′ E, and is surrounded by the mountains. The total area of Beijing is 16,410.54 km^2^, divided into 16 administrative districts with an estimated population of 21.7 million in 2017 [16]. Using geographic information systems (GIS), TB cases were geo-coded to the neighborhood (district) level, and surveillance records were examined to avoid misclassification. Likewise, spatial regression models were utilized to observe the relationship between TB incidence and socio-economic as well as health facility parameters.

### 2.2. Data Collection

Tuberculosis (TB) cases were the outcome variable of this study. Data on all reported cases of tuberculosis (including age, sex, address, and clinical records) between 2005 and 2014, were obtained from Beijing Information of Tuberculosis Prevention and Control registration database [25]. Predictor variables of each district including geographical, socio-economic, and health service factors such as: number of health institutes (NHI; number of hospitals or health centers in the study area), number of hospital beds (NHB; number of beds in the hospitals in the study location), per capita GDP (PC_GDP/USD), population density/km (PD/km^3^ population density for each central area of a district within a 3 km surrounding area), county/district level GDP (C_GDP) (each district’s GDP), and permanent resident population (PRP) were collected from the Beijing Statistical Yearbook, covering the years from 2005 to 2014 [16]. These variables are well known influencing factors for TB, and were therefore selected to fulfil our objectives. 

All collected data were imported into Microsoft Excel 2010 (Microsoft, Redwoods, WA, USA) and linked to the respective location in the geographic information system. To conduct a GIS-based analysis of the spatial distribution of TB, district/county level polygon maps at 1:100,000 scales were obtained from the National Geometric Center of China [26], and a district level polygon layer map including the information regarding latitudes and longitude of the central points of each county with an attributes table was created. Patients information, including district information, was geo-coded utilizing ArcGIS Info Software V. 10.0.1 (Environmental Systems Research Institute, Redlands, CA, USA) [27]. In addition, after geocoding, TB cases were matched automatically and interactively with their respective residential addresses.

#### Ethical Approval

We obtained ethical approval and clearance from the ethical review committee of Capital Medical University (SPHCMU), Approval No. IRB00009511, Beijing, China and a consent from each individual subject was not required because we used only aggregated data from the Beijing Center for Disease Control (CDC, Beijing, China).

### 2.3. Statistical Analysis

#### 2.3.1. Descriptive Analysis

All descriptive information of variables was summarized annually according to geographic area (districts), where incidence rates were calculated for each district using neighborhood census data as a denominator and reported TB cases as the numerator. 

#### 2.3.2. Spatial Analysis

We used the ArcGIS tools to generate the Moran’s *I* index value and *Z*-score, including the *p*-value, which evaluated the significance of that index. These values assess whether the distribution pattern is clustered, dispersed, or random. This method permits a test for the existence of global heterogeneity, in terms of spatial autocorrelation or spatial dependency [28]. Spatial autocorrelation (Global Moran’s *I*) was used to investigate the presence of spatial as well as clusters of TB cases and to identify their possible locations. The calculation is based on the features’ locations as well as their attribute values [29]. 

The Moran’s *I* statistic for spatial autocorrelation is given as:

$$I=\frac{n}{S_{o}}\frac{\sum_{i=1}^{n}\sum_{j=1}^{n}\omega_{i,j}z_{i}z_{j}}{\sum_{i=1}^{n}z_{i}^{2}}$$

where, $z_{i}$ is the deviation of an attribute for featuring *i* from its mean ($X_{i}-\bar{X}$), $\omega_{i,j}$ is the spatial weight matrix between feature *i*, and *j*, *n* is equal to the total number of features, and $S_{o}$ is aggregate of all the spatial weights:

$$S_{o}=\sum_{i=1}^{n}\sum_{j=1}^{n}\omega_{i,j}$$

The *z_I_*—score for the statistic is computed as:

$$z_{I}=\frac{I-E\left[I\right]}{\sqrt{V\left[I\right]}}$$

where

$$\begin{array}{c} E\left[I\right]=-1/\left(n-1\right)\\ V\left[I\right]=E\left[I^{2}\right]-E{\left[I\right]}^{2} \end{array}$$

where, Moran’s *I* index, *Z* scores, and *p*-values were used to evaluate the significance of the test. A Global Moran’s *I* Index value near +1.0 indicates the clustering, which means the TB incidence should be similar among the neighboring districts, while a value near −1.0 indicates dispersion and zero means total spatial randomness. The absolute value of global Moran’s *I* value specifies the strength of the spatial autocorrelation [28,29]. 

#### 2.3.3. Correlation Coefficient Analysis

The Spearman correlation coefficient was used to analyze the correlation between TB incidence and predictor variables in Beijing districts. This is a parametric measure, that measures the non-linear distributed pattern of the variables of the data [30]. The coefficient ranges from −1 to +1. If the correlation coefficient approaches zero, there is a weak correlation between the variables, where near to +1 or −1 the correlation between two variables is stronger. The reason for using the correlation coefficient method was to provide a complete information on the association between TB incidence and affecting factors (socio-economic). The correlation estimate was performed using SPSS version-20 (SPSS Statistics 20, IBM: Corporation 1 New Orchard Road, Armonk, NY, USA). 

#### 2.3.4. Spatial Regression Analysis

The study assumes that the tuberculosis infection is influenced by several factors, such as demographic factors (resident population, population density, migrate population), economic factors (per capita GDP, district- level GDP), and health facility indicators (number of beds in hospitals, number of health institutes). Therefore, we analyzed the average values that were used to explore the predictors of TB incidence and increase the stability of data and minimize the potential bias [31]. The average incidences of TB at district level over the 10 years study period were also calculated.

Then, we constructed the shapefile of the outcome and predictor variables in ArcGIS software and exported it into the GeoDa (The Open GeoDa environment 1.8.6 (Luc Anselin, Phonix, AZ, USA) for advanced geospatial analysis. GeoDa is the latest spatial data analysis software tool developed by the Center for Spatially Integrated Social Sciences (CSISS) to apply various types of exploratory spatial data analysis including data manipulation, mapping, and spatial regression analysis. We generated a spatial weights matrix in GeoDa software, which is necessary for the calculation of spatial autocorrelation statistics. Basically, spatial weights can be constructed in both ways; either based on contiguity from polygon map files or based on the distance between points. We selected contiguity based on spatial weights since our main interest lies in understanding the spatial interdependence between the outcome variable and a set of exposure variables in the nearby districts. GeoDa further provides two types of spatially contiguous weights, that is, rook’s weight (using common boundaries to define the neighbor), and queen’s weight (including all common points that are boundaries and vertices). Finally, we used rook’s contiguity (Figure S1) weight for estimating all geospatial statistics and geospatial regressions [32].

Firstly, we employed the classic ordinary least square (OLS) model to estimate the effect of various socio-economic and health facility variables upon the outcome variable. After learning about the presence of spatial dependence in the outcome and predictor variables, we understood that the assumption of independent observations and errors of this model might be violated. Thus, we employed and compared three regression models: ordinary least square (OLS), the spatial lag model (SLM) [33] and the spatial error model (SEM) [34] to examine the relationship between the outcome variable with a set of predictors. Spatial regression methods estimate spatial dependency in regression analysis, avoiding statistical problems such as unstable parameters and unreliable significance tests, and provide information on spatial relationships among the parameters involved in the model [33,34,35]. The OLS regression model takes the following form:

y= ∝+βx+ε

The spatial lag model (spatial auto-regressive model) has the following form:

y= ∝+ρWγ+βx+ε

Then, the spatial error model has the following form:

y= ∝+ρWγ+βx+ε, with ε=λWε+ζ

where y denotes TB incidence, ∝ is an intercept, β is the vector of regression parameters, x is the matrix of exogenous explanatory variables, *ε* is the vector of regression disturbances, Wγ the spatial lag term, ρ is the spatial autoregressive parameter of Wγ (which is estimated for the model as a whole), and λ is the coefficient of spatially lagged autoregressive errors, Wε, Errors in ζ are independently distributed, and *W* is the spatial weight [33,34,35].

## 3. Results

### 3.1. Distribution and Trend of Tuberculosis Incidence

Of a total of 44,408 new sputum smear-positive pulmonary tuberculosis cases of Beijing districts from 2005 to 2014, we used 40,878 cases and the remaining 3530 cases were excluded due to unavailable resident information or because those patients did not reside in the Beijing region. Of the total 26,713 patients with TB, 44.1% (19,601) were aged between 16 and 30 years old, while 34.2% (15,170) were aged between 31 and 60 years old (a productive age group). In addition, 12.8% (5705) were aged over 61 years and only 0.9% (402) were aged under 6 years. 

**Figure 1:**
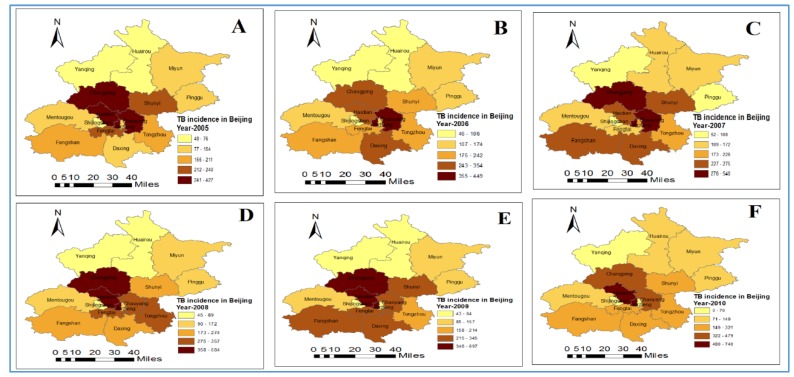

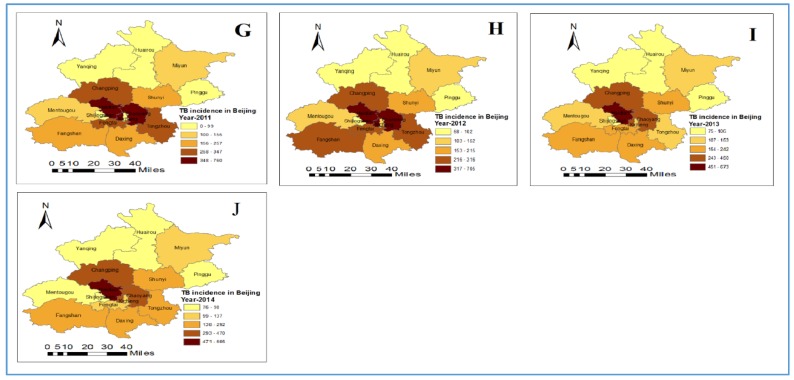
Trends of pulmonary tuberculosis (TB) incidence in Beijing districts, from 2005–2014 as respective (**A**–**J**) in the figure.

A remarkable reducing trend in TB incidences was noted in the Beijing region from 2009 to 2014 (Figure 1). A higher incidence of TB was found in densely populated districts in Beijing, for example: Haidian (urban function-extended district) and Xicheng. TB incidence rates were noted as high in the Xicheng and Mentougou (ecological preservation, development, and core district of capital function) districts during the whole study period (Figure 1).

### 3.2. Description of Variables

**Figure 2:**
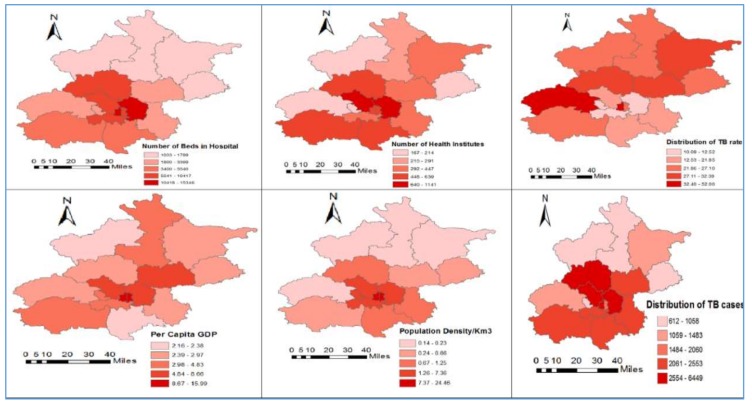

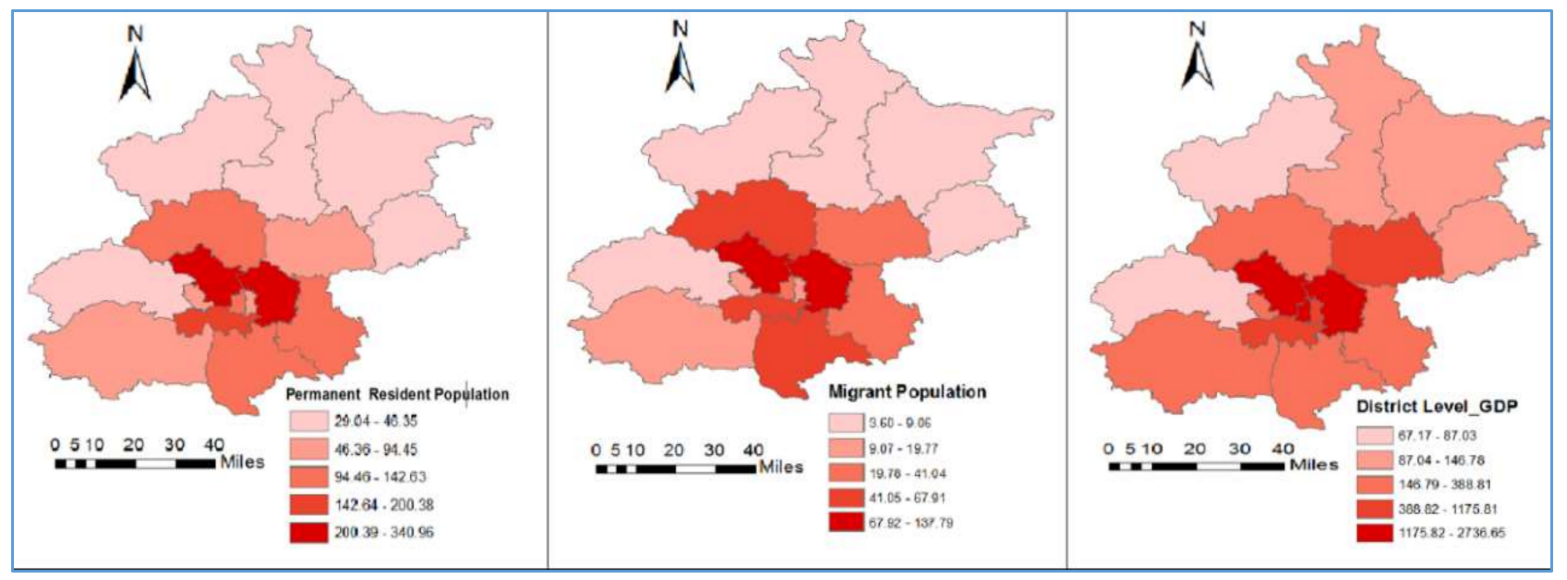
Description of outcome and predictor variables during the study period.

Table 1 describes the descriptive statistics for each of the selected variables, including the Global Moran’s *I* index with significant values (*p* = 0.05 level). The outcome variable, pulmonary TB (total 40,878 cases) ranged from 39 to 885 (mean/SD) = 256.37/183.34). All descriptive information of the variables, including the maximum, minimum, mean, standard deviation and Global Moran’s *I* with significant values are displayed in Table 1 and Figure 2.

### 3.3. Spatial Pattern of Tuberculosis Incidence

In order to magnitude the geo-spatial autocorrelation and clusters of the outcome variable, we applied the Global Moran’s *I* statistics for each study year separately. The global spatial correlation analysis exhibited the presence of positive spatial clustering of TB incidence in the Beijing region (*Z*-score from 0.374 to 1.741) (Table 2). 

### 3.4. Correlation Coefficient Analysis

The Spearman correlation coefficient analysis is applied to estimate the relationship between tuberculosis incidence and other independent variables. A significant positive correlation found between TB incidences and socio-economic predictors (at the 0.01 level in the two-tailed test of significance) (Table S1). 

### 3.5. Spatial Regression Analysis

In order to select an appropriate spatial regression model from spatial regression models, the Lagrange multiplier (LM) diagnostics tests were employed to determine spatial dependence. Two spatial weights matrices (raw standardized and distance-based spatial weights) were used for this diagnostic test to make sure the results were robust or not (Table S2). As for TB, both the LM lag and LM error test statistics including Moran’s *I* and LM (SHRMA) tests were highly significant in both weights matrices, except for the robust LM lag (0.0375, *p* > 0.846), indicating that there a strong spatial dependence existed. Therefore, the robust LM test was applied for both the SLM and SEM. 

#### Statistical Analysis of Residuals from OLS, SLM and SEM

First, we applied OLS models to fit the model under the classical Best Linear Unbiased Estimator (BLUE) assumptions. We found a significant spatial clustering of the outcome and exposure parameters in the OLS models. Moran’s *I* score of −0.0631 (*p* < 0.001) was highly significant, indicating a strong spatial autocorrelation of the residuals. Additionally, both Lagrange multiplier (lag) and Lagrange multiplier (error) test results were significant, expect the Robust LM (lag); indicating the presence of spatial dependence. The robust estimation of the error term (7.915; *p <* 0.001) is still significant, however, the robust lag test (0.0375; *p* = 0.846) is insignificant, which means that when a lagged dependent variable is present, the error dependence disappears. The coefficient of determination (R^2^) of the OLS model was (0.359), indicating that the risk factors during study period explained 35.9% of the total variance for TB incidence, also indicating that the OLS model was not the best fit for this group of data. From the estimation of the OLS model, four variables were found to be statistically significant with respect to TB incidence. Higher numbers of beds in the hospital and per capita GDP had a positive effect on TB incidence, while population density per 3 -km range from the center of the district and higher migrant population had negative effects on TB incidence (Table 3).

After identifying the presence of spatial dependency on the OLS model, we further re-estimated the spatial regression model (SLM and SEM) with the maximum likelihood estimation approach while controlling for the spatial dependency. We determined the spatial lag term of TB with the designed spatial weight file. The coefficient parameter (Rho ‘ρ’) revealed the spatial dependence inherent in our sample data in that measuring the average influenced the observations of their neighboring observations. The determination coefficient (R^2^) was 0.3975, indicating that the model explained 39.75% of the variance in TB incidence. The decreasing values of log-Likelihood and AIC and the increasing value of R^2^ also suggested here a general improvement of the model fit in the SLM as compared to the OLS. This SLM results showed that numbers of beds in hospital and district-level GDP had a positive relationship, while per capita GDP, population density/3 km, permanent resident population, and migrat population had negative significant associations with TB incidence. Likewise, we extended the SEM to estimate the relationship between the exposures and the outcome variable. The spatial error parameter (λ) was statistically significant, as indicated by the *p*-value (<0.001). In SEM, the R^2^ value was found to be 0.4132, indicating that the model explained 41.32% of the variance in TB incidence. 

After comparing the results of the R^2^, AIC, and log likelihood test of regression diagnostics, the general model fit was improved as compared to the other two models (OLS and SLM). The Breusch–Pagan test (16.44; *p* = 0.021) and the likelihood ratio test (18.30; *p* < 0.001) of spatial error dependence are still significant, which means that the spatial effects in the data were still not removed completely. However, both spatial models yield improvement than OLS model and the spatial error model appeared to fit better than the SLM. In SEM, the number of beds in the hospital and per capita GDP was significantly and positively associated with TB incidence, while district- level GDP, population density/3 km, and migrant population were significantly and negatively associated with TB incidence during the study period. However, per capita GDP was no longer negatively associated with TB incidence in the SLM model and showed a positive correlation. The number of health institutes had a positive correlation, while the permanent resident population had an adverse association with TB incidence during the same study period in SLM. However, these variable coefficients were not significant (Table 3). 

## 4. Discussion

Using GIS technology, this study is the first attempt to estimate the factors associated with TB incidence, taking into account with socio-demographic and health facilities variables at the district level in Beijing. Previously studies have reported that many factors such as GDP per capita, district -level GDP, available health facilities with proper beds, population density, and migrant population may influence TB infection [7,21,36]. Our study found that all predictors were statistically significant in the Spearman correlation coefficient analysis (Table S1). We used consistent spatial regression models to forecast the relationship between TB incidence and predictor variables, and the results of the SEM suggested that the number of beds in hospitals, per capita GDP, population density, and migrant population were significantly correlated with TB incidence in the study location.

Recent evidence from the previous studies has highlighted the correlation between TB incidence and health indicator/ demographic factors as well as socio-economic factors [13,20,37,38,39]. However, we did not find a strong relationship between these variables and TB incidence in our study, except for the health facility indicators. Our study supports the view that health institutes or health care facilities may affect TB incidence. This influence can be maximized through efforts by the individual and community, or at socio-economic and geographic levels by providing education or knowledge (health awareness) towards TB prevention and control, making sure acceptable and assessable health services are available, and improving household income, wealth, and other associated factors [1,2,21]. 

Furthermore, our finding suggests that the population density per 3 km distance (from the center point of the district headquarter), permanent resident population, and migrant population had a negative correlation with TB incidence, however, those factors were positively associated with TB incidence as reported by previous studies of Sun, et al. [13] and Liu, et al. [21]. This difference could be due to study duration, as we used different periods of time for data in the same location. Another reason could be that Beijing is a capital city of China, where more facilities are available compared to other cities of China. Such facilities including educational institutes (schools, colleges and universities) and health institutes (hospitals, medical colleges, health centers, clinics and TB treatment centers) which enable an increase the awareness level of the people toward TB disease. Economic status in Beijing is another factor, as only economically sound people can survive in the city like Beijing, where each and every type of health care service is easily available in the government-owned or private sectors. Notably, migrant population and population density highly influence risk factors in Beijing, as revealed in the earlier studies [7,13]. One study revealed that migrants were 13 times more likely to be associated with TB infection compared to settled population [40]. Likewise, a previous study experienced that migration and population density were significant risk factors for TB infection [41]. A few years back, the prevention and control of TB infection among the migrant population posed a great challenge in Beijing, where the migrant population was rapidly increasing [17,42]. Moreover, the prevalence of sputum smear-positive TB in rural areas was 1.6 times higher than in urban areas in China, as estimated by National TB Prevalence Survey in 2010 [43]. Similar trends were found in Africa [44], Pacific Island countries [45], and Australia [46]. In fact, it is already well-established that TB incidence is much higher in rural settings compared to the urban cities in developing countries [1,2,47]. Therefore, there should be an emphasis on access to the health care facilities, as there is better access to the health care facilities in the urban areas. 

In our study, health care facility indicators have had a weakly positive association with TB incidence. In contrast, a negative impact was found in the study of Sun, et al. [13]. The explanation behind this could be that our study was only based on the Beijing region (16 districts), whereas Sun, et al.’s study covered all geographical areas of China [13]. A good quality and accessible health care system always results in better health outcomes of that country. Hospital and other healthcare settings are of benefit to TB patients. Because treatment of TB is quite expensive and long-term, patients should have access to a free treatment service provided by the government through coordination with WHO.

Another finding of this study is the association between TB incidence and economic factors. The correlation of TB with per capita income was found to be insufficient relationship, while it a positive relationship was found with respect to with district-level GDP, indicating that the economic status of the individual as well as the overall county level is an important factor for improving the health status. Notably, it has already been established that TB is a poverty-related disease [36] so there is no need to explicitly explain the relationship of per capita GDP with the occurrence of TB. In fact, wealth indices have been found to be significantly associated with TB infection in Zambia and South Africa too [48]. Consistent findings have been found in the previous studies [21,36], which means that if the per capita GDP increases, TB incidence decreases, confirming that lower economic status is a risk predictor for TB incidence. In recent years, China has experienced a rapid economic development and urbanization. The average per capita GDP of Beijing was recorded at 5.522 per US $1000, whereas at district level the GDP was US $771.3288 billion during our study period. At the national level, per capita GDP was recorded to be US $6416.18 in 2015 with an average of US $1453.86. In 1962, the per capita GDP was only US $130.14 in China, according to the World Bank [49]. Tuberculosis control projects have successfully accomplished their objective; however, TB remains a major long-term public health burden problem in China. Recently, a significant contribution towards health financing has been implemented in China with the support of The World Bank [49], alongside a TB control program and other public health programs, these being systemic reforms rather than isolated public health interventions in the country. 

Although this study was a spatial analysis of socio-economic predictors and distribution of tuberculosis in the Beijing region, there are some limitations which should be acknowledged. First, under-reporting is an issue for any infectious disease surveillance system in China, like other countries. We extracted TB data from the official surveillance system, where cases might be missed through the routine notification system. Second, this study did not include several factors related to pulmonary TB incidence due to unavailability of the information at the district level, such as climate factors (annual average temperature, annual precipitation, relative humidity, rainfall and sunshine hour), health-related factors (death rate, number of doctors per/10,000 individuals in the population), and economic factors (unemployment, household income). The epidemiological study focuses on both time and space, which influence the disease occurrence. Therefore, a study focusing on a spatiotemporal analysis along with the correlation with environmental factors and TB incidence is urgently needed.

## 5. Conclusions

This study investigated socio-economic predictors and distribution of smear-positive tuberculosis in the Beijing region. We found that TB incidence mostly occurred in urban and densely populated districts over the study period. However, the trend of TB incidence has been decreasing in Beijing. Consistently, we confirmed that the number of the hospital beds, district-level GDP, per capita GDP, permanent resident population, population density, and migrant population may have an impact on TB incidence. Our findings suggest that TB control measures should be focused on those factors in order to allocate public health resources more precisely to reduce the burden of TB incidence. 

## Figures and Tables

**Table 1:** Description of outcome and predictor variables.

Variables	Min	Max	Mean	Std. Deviation	Moran’s *I*	*p*-Value
Tuberculosis cases (TBC)	39	885	256.37	183.340	0.193	<0.001
Tuberculosis rate (TBR)	5.96	71.78	25.8218	12.97541	−0.010	0.446
Number of health institutes (NHI)	68	1337	472.40	310.911	0.210	<0.001
Number of hospital beds (NHB)	982	19,053	5763.34	4498.153	0.422	<0.001
Migrant Population (M_P)	2.0	180	38.90	42.045	0.264	<0.001
Per capita GDP (PC_GDP)	1.38	23.44	5.5224	4.49726	0.254	<0.001
Population density per 3 km (PD_3)	0.13	25.55	4.9954	7.35956	0.439	<0.001
Permanent resident ropulation (PRP)	27.7	392.2	117.259	94.4632	0.298	<0.001
County/district level GDP (C_GDP)	0.40	43.37	7.8275	9.84348	0.305	<0.001

County/district level gross domestic product (C_GDP) expressed in Billions of US dollars, per capita GDP (PC_GDP) = expressed in thousands of US dollars.

**Table 2:** Global spatial autocorrelation analysis of TB incidence in Beijing, 2005–2014.

Years	Moran’s-*I*	*Z* Score	*p-*Value
2005	0.199	1.920	0.033
2006	0.177	1.741	0.045
2007	0.118	1.627	0.059
2008	−0.012	0.374	0.326
2009	−0.009	0.419	0.319
2010	0.062	0.897	0.168
2011	0.123	1.326	0.104
2012	0.163	1.685	0.051
2013	0.100	1.354	0.107
2014	0.104	1.142	0.126

**Table 3:** Results of the Ordinary least square (OLS) model, the Spatial lag Model (SLM) and the spatial error model (SEM) assessing the correlates of the TB rate with maximum likelihood estimation.

Variable	Ordinary Least Squares Model				Spatial Lag Model				Spatial Error Model			
	Coefficient	St.Error	T-Value	*p*-Value	Coefficient	St-Error	Z-Value	*p*-Value	Coefficient	St-Error	Z-Value	*p*-Value
NHI	−0.00528	0.00583	−0.9058	0.3664	−0.005624	0.00551	−1.0197	0.307	0.002067	0.0073	0.27953	0.779
NHB	0.00332	0.00065	5.11351	**<0.001**	0.003137	0.00061	5.08384	**<0.001**	0.004666	0.00060	7.7630	**<0.001**
C_GDP	0.71719	0.28287	2.53537	**0.012**	0.858366	0.27062	3.17185	**0.001**	−0.131481	0.32594	−0.4033	0.686
**PC_GDP**	−0.90688	0.53734	−1.6876	0.093	−1.081062	0.51153	−2.1133	**0.034**	1.349843	0.60574	2.22839	**0.025**
PD_3	−0.73422	0.32684	−2.2463	**0.026**	−0.69715	0.30916	−2.2549	**0.024**	−2.209779	0.33873	−6.5236	**<0.001**
PRP	−0.08174	0.04392	−1.8609	0.064	−0.099438	0.04152	−2.3944	**0.016**	−0.017595	0.04182	−0.4206	0.673
M_P	−0.26147	0.08852	−2.9537	**0.003**	−0.248568	0.08481	−2.9307	**0.003**	−0.371144	0.09248	−4.0130	**<0.001**
Lambda (λ)									10.57686	1.54012	6.8675	**<0.001**
Rho (ρ)					−0.795591							
R^2^	0.359035				0.397502				0.413278			
Log-likelihood	−601.035				−594.763				−591.880			
AIC	1218.07				1207.53				1199.76			
BPT	58.9438			**<0.001**	59.9531			**<0.001**	16.4445			**0.0213**
LRT					12.5443			**<0.001**	18.3090			**<0.001**

TBC = tuberculosis cases, TBR = tuberculosis rate, NHI = number of health institutes, NHB = number of beds in the hospital, M_P = migrant population, PC_GDP = per capita GDP, PD_3 = population density/3 km, PRP = permanent resident population, BPT = Breusch–Pagan test, AIC = Akaike information criterion, LRT = likelihood ratio test, Significant is at the *p* < 0.05 level.

## Supplementary Materials

The following are available online at http://www.mdpi.com/2076-3271/6/2/26/s1, Table S1: Spearman correlation coefficient Matrix of all Variables, Table S2: Diagnostic test for spatial dependency, Figure S1: Spatial contiguity weights: rooks and queens. (A. Rook’s Weight. B. Queens’s Weight).
