# Supplementary material for: Socio-Economic Predictors and Distribution of Tuberculosis Incidence in Beijing, China: A Study Using a Combination of Spatial Statistics and GIS Technology
Source: Med Sci (Basel). 2018 Mar 21;6(2):26. doi: 10.3390/medsci6020026 (PMC6024827; doi:10.3390/medsci6020026)
Supplement: Supplementary file 1 [file medsci-06-00026-s001.pdf]

Socio-Economic Predictors and Distribution of Tuberculosis Incidence in Beijing, China: A Study Using a Combination of Spatial Statistics and GIS Technology

Supplementary Table 1. Spearman Correlation Coefficient Matrix of all Variables

	Correlations							
**Variables**	**1**	**2**	**3**	**4**	**5**	**6**	**7**	**8**
1. Total Tuberculosis cases (TBC)	1.000							
2. Number of Health Institute (NHI)	.673**	1.000						
3. Number of Hospital Beds (NHB)	.755**	.740**	1.000					
4. Migrant Population (M_P)	.728**	.774**	.777**	1.000				
5. Per Capita GDP (PC_GDP)	.333**	.586**	.633**	.414**	1.000			
6. Population Density (per 3 Km)	.586**	.568**	.874**	.706**	.667**	1.000		
7. Permanent Resident Population (PRP)	.789**	.799**	.820**	.952**	.410**	.758**	1.000	
8. County/ district level GDP (C_GDP)	.687**	.794**	.889**	.823**	.796**	.877**	.851**	1.000

**. Correlation is significant at the 0.01 level (2-tailed).

Supplementary Table 2. Diagnostics for result for the Spatial Dependence

**Type of Test**	**Test to determine the inclusion of the model with spatially lagged or spatial error auto correlated term**					
	Moran's I (error)	LM (SARMA)	LM (lag)	Robust LM (lag)	LM (Error)	Robust LM (error)
Statistics Value	-5.6573	16.6951	8.7799	0.0375	16.6576	7.9152
P -Value	**<0.001**	**0.00024**	**0.00305**	0.84647	**0.00004**	**0.0049**

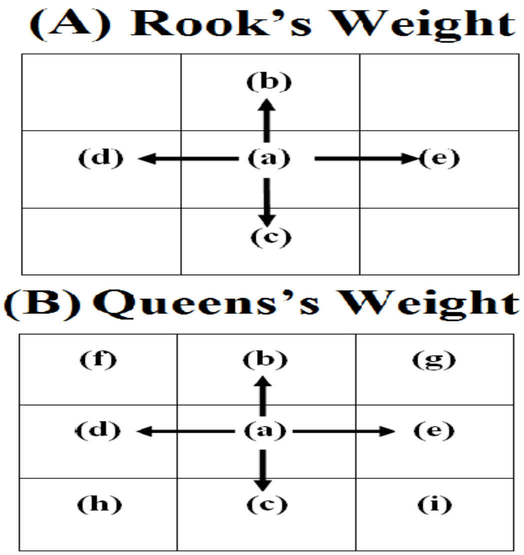

S. Fig 1. Spatial contiguity weights: Rooks and Queens. A. Rook's Weight. B. Queens's Weight
